# Novel Ultrasound Anatomical Measurement of the Deep Transverse Metatarsal Ligament: An Intra-Rater Reliability and Inter-Rater Concordance Study

**DOI:** 10.3390/jcm11092553

**Published:** 2022-05-02

**Authors:** María del Mar Ruiz-Herrera, Félix Marcos-Tejedor, Alberto Aldana-Caballero, César Calvo-Lobo, David Rodriguez-Sanz, Simone Moroni, Marko Konschake, Alicia Mohedano-Moriano, Javier Aceituno-Gómez, Juan José Criado-Álvarez

**Affiliations:** 1 María del Mar Ruiz Clinic, 13600 Alcázar de San Juan, Ciudad Real, Spain; clinicamariadelmarruiz@gmail.com; 2 Department of Medical Sciences, Faculty of Health Sciences, University of Castilla-La Mancha, 45600 Talavera de la Reina, Toledo, Spain; alicia.mohedano@uclm.es (A.M.-M.); jjcriado@jccm.es (J.J.C.-Á.); 3 Department of Nursing, Physiotherapy and Occupational Therapy, Faculty of Health Sciences, University of Castilla-La Mancha, 45600 Talavera de la Reina, Toledo, Spain; alberto.aldana@uclm.es; 4 Faculty of Nursing, Physioterapy and Podiatry, Complutense University of Madrid, 28040 Madrid, Spain; cescalvo@ucm.es (C.C.-L.); davidrodriguezsanz@ucm.es (D.R.-S.); 5 Department of Podiatry, Faculty of Health Sciences at Manresa, University of Vic-Central University of Catalunya (UVic-Ucc), 08500 Vic, Barcelona, Spain; dott.simonemoroni@gmail.com; 6 Department of Anatomy, Histology and Embryology, Institute of Clinical and Functional Anatomy, Medical University of Innsbruck (MUI), 6020 Innsbruck, Austria; marko.konschake@i-med.ac.at; 7 Management of Integrated Assistance of Talavera de la Reina, Health Service of Castilla-La Mancha, 45600 Talavera de la Reina, Toledo, Spain; javier.aceituno@uclm.es; 8 Department of Nursing, Physiotherapy and Occupational Therapy, Faculty of Nursing and Physiotherapy, University of Castilla-La Mancha, 45600 Talavera de la Reina, Toledo, Spain; 9 Department of Health, Institute of Health Sciences, 45600 Talavera de la Reina, Toledo, Spain

**Keywords:** Morton’s Neuroma, deep transverse metatarsal ligament, ultrasound, concordance

## Abstract

Insufficient space below the Deep Transverse Metatarsal Ligament (DTML) could be an etiological factor for Morton’s Neuroma (MN). To date, there is a lack of studies measuring the space below the DTML. For this reason, this study assesses the intra- and inter-rater concordance and reproducibility of measurements of the space below the DTML between the third and the fourth metatarsal heads (M3 and M4) using ultrasound imaging to assess and verify the reliability and reproducibility of measurements of the space under the DTML. Forty feet from twenty patients were examined using ultrasound by three trained evaluators at two different times. The two measurements taken on each foot were: base (b)—distance between M3 and M4, and height (h)—distance between the DTML and the plantar skin surface. This was a quantitative, observational, analytical study. The concordance rate between observers for measurements of height and base were 98.5% and 99.5%, respectively. The mean area obtained of the space was 54.6 mm^2^ and 57.2 mm^2^ for both the left and right foot (*p* > 0.05). Reproducibility over time calculated in pre- and post-measurements showed an intraclass correlation coefficient of 1.00 (95%CI: 0.99–1.00), which leads us to conclude that the measurements are perfectly reproducible. Both measurements (height and base) of the space under the DTML, performed by ultrasound, are reliable and reproducible.

## 1. Introduction

Insufficient space under the Deep Transverse Metatarsal Ligament (DTML) can be linked to the development of Morton’s Neuroma (MN) [1]. The etiology of MN remains unclear, although it is a frequent condition found in clinical practice, and its onset is one of the most important causes of pain in the forefoot [2,3]. The compressive theory is the most accepted, in which compression forces produced in the tunnel between adjacent metatarsals, the DTML, and the surface of the ground compress the neurovascular bundle and generate MN. This pressure on the surrounding soft tissues has been suggested as the cause of the secondary changes produced in the common digital nerve, and leads to MN. The increase in pressure is the result of a decrease in space in the study area, due to anatomical, structural, and/or biomechanical factors, such as equinus or activities that overload the forefoot [1,2,3,4,5].

Ultrasound (US) measurements of the space below the DTML are a way of assessing the anatomy of the area between the third and the fourth metatarsal heads (M3, M4), and, therefore, it is important to establish clear references following studies to determine the reliability and concordance between US measurements [4]. An assessment of the anatomy of the surrounding structures related to the MN, including DTML, is needed [5].

The aim of this study is to assess the intraevaluator reliability, interevaluator concordance, and intraevaluator repeatability of the measurements of the space under the DTML using ultrasound imaging.

## 2. Materials and Methods

The present study received a favorable opinion from the Clinical Research Ethics Committee of the University Rey Juan Carlos (Alcorcón, Madrid) under the registration number 1312201900320. Participants signed an informed consent form and provided demographical information.

The study was designed to determine the intra- and interevaluator concordance between measurements with US [4], between the plantar skin and the DTML, in the middle area between M3 and M4. The updated list of essential elements for studies of diagnostic precision was followed [6].

Twenty patients (forty feet) attending the María del Mar Clinic were recruited between February and April 2020. After calculating the minimum sample size needed to detect a correlation coefficient significantly different from zero [7] for a minimum value of 0.7, with a 95% confidence interval, a bilateral type I error of 5% (Risk α = 0.05), with a power set at 80% (type II error, β = 0.2), a minimum size of 14 patients is obtained. Considering a maximum loss of subjects of 30%, the number of participants needed is 20, to whom a complete measurement of both feet will be performed, with a total of 40 complete measurements.

Participants were required to be between 18 and 65 years of age. Exclusion criteria included [5,8,9] solution of continuity in plantar skin, presence of ulcers and/or blisters, having undergone foot surgery, congenital or acquired malformations, pregnancy, keratotic lesion on the plantar surface, exercise 48 h before exploration, use of high heels 48 h before exploration, equinus, biomechanical alterations, impossibility to visualize the structures with US, structural alteration of the transverse arch of the metatarsals, rheumatic conditions, and metatarsalgia.

The intraevaluator reliability assessment required the participant to be seated for 15 min before the exploration. During that time, the objectives and the exploration procedure were explained to the subjects, and, after verifying understanding, informed consent was obtained. The exploration was performed with the participant seated, barefoot, and feet hanging from the ankles. To prevent the participant from contracting the muscles and altering the measurement, the researcher checked for abduction and mild plantar flexion, indicating muscles were relaxed [9].

**Figure 1:**
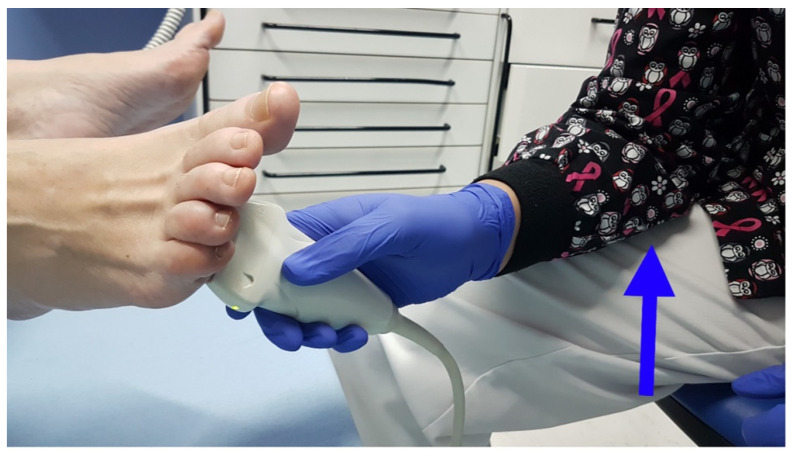
The evaluator will be seated, resting their arm on their leg (blue arrow) for stability, and will not touch the patient’s foot.

**Figure 2:**
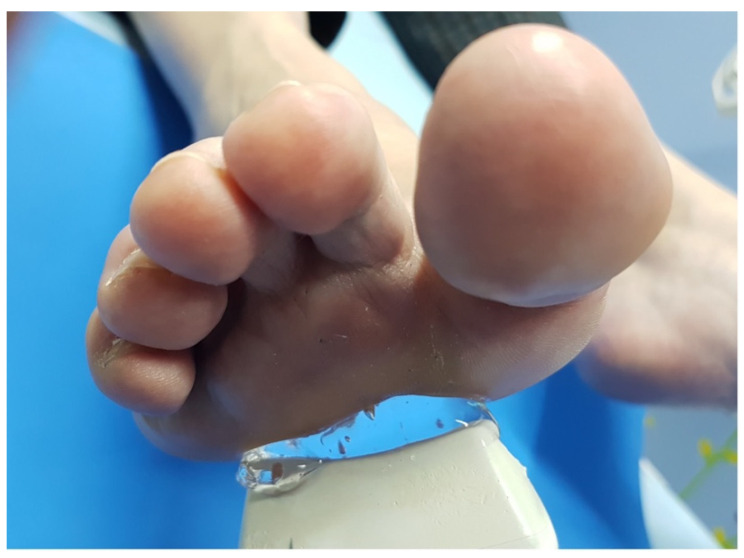
A generous amount of gel was applied between the skin and the probe, without adding pressure or touching the skin of the participant with the probe.

**Figure 3:**
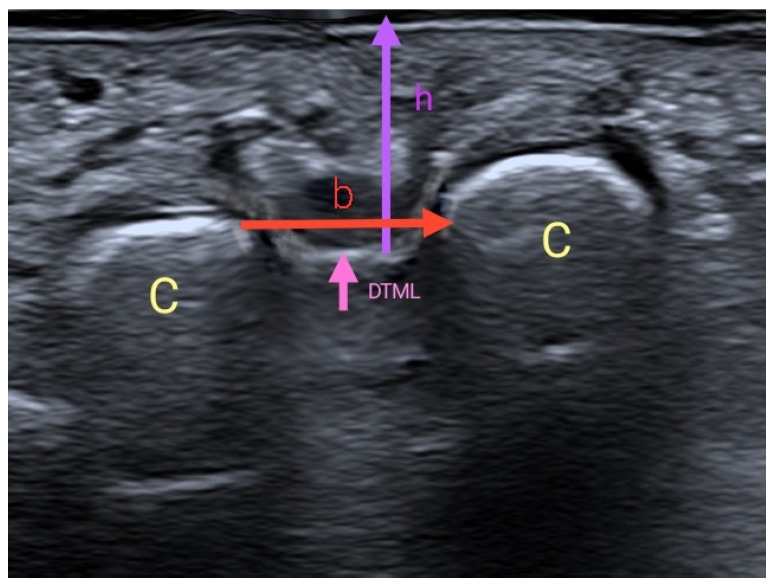
Ultrasound imaging of the measurements. C: Metatarsal heads 3 and 4 (M3 and M4). DTML: Deep transverse metatarsal ligament. h: Height: Distance between the DTML and the plantar skin in the middle area between M3 and M4. b: Base: Distance between M3 and M4.

To assess the interevaluator concordance, the two US measurements were taken consecutively by three trained evaluators, with at least 2 years of experience. The evaluator was seated, resting the arm which held the US probe on their own leg, to avoid pressing the skin of the participant (Figure 1) [10]. A generous amount of gel was applied between the skin and the probe, without adding pressure or touching the skin of the participant with the probe (Figure 2). The image was taken with the probe perpendicular to the skin without touching it, once the DTML and M3 and M4 heads of the foot under exploration were located. Two measurements were taken with US [11]: a vertical measurement or height (h), between the plantar skin and the DTML in millimeters; and a horizontal measurement or base (b), between the area closer to the M3 and M4 heads. With these two measurements, a rectangular area was obtained (A = b*h) in square millimeters (Figure 3). Both measurements (h and b) were taken in each foot in each participant by three different evaluators in consecutive times (Pre and Post), with a resting time of 30 min in between. Therefore, each participant was evaluated 6 times in each foot (12 measurements per subject).

During each exploration, the other two evaluators checked for possible errors of the evaluator performing the exploration [12]. These evaluators were not allowed to visualize the measurements taken (assessor blinding). Later, the same measurements were taken immediately by the other two evaluators (interevaluator reliability). Each step of the measurement protocol was evaluated in a dichotomous way (0 = non-compliance of the step, 1 = compliance of the step) by the two evaluators simultaneously and independently, obtaining two scores for each of the 12 measurements taken on each participant. In order to standardize the measuring mode, a rigorously observed protocol consisting of a 10-step check list was designed: (1) Is the participant relaxed? (foot in slight abduction and plantar flexion)? (2) Does the evaluator taking the measurements check step 1? (3) Has the participant understood and signed informed consent? (4) Is the probe separated from the skin without exerting pressure? (5) Does the evaluator taking the measurements have the arm resting on their leg? (6) Is the area of exploration correct? (7) Is the position of the probe correct? (8) Are they sure about the measurement taken? (9) Do they record the measurement? (10) Are the two evaluators recording the exploration on their evaluator’s annex?

The value is obtained by adding the scores given to each of the 10 steps. The order of performing the exploration to participants by the evaluators and the order of the foot under exploration were randomized.

The US images were recorded using a high-resolution digital ultrasound diagnostic system, Esaote MylabFive (Esaote Europe BV) with a Bisound-Esaote LA435 type linear transducer with a frequency between 6 and 18 MHz bandwidth.

The statistical analysis was made using SPSS 19.0 for Windows. In the descriptive statistical analysis, the parameters used depended on the variable of study. The Shapiro–Wilk test was used to determine variable distribution. The inferential statistical analysis of independent variables depended on the scale of each variable. The intraevaluator reliability was assessed using Wilcoxon ranks test. The interevaluator concordance was calculated with the intraclass correlation coefficient with 95% confidence intervals (95% CI) [13]. A type I error of 5% was considered [14].

## 3. Results

A total of 20 patients participated in this study, of which 13 (65%) were women, with a mean age of 40.6 ± 6.21 years. The mean time for evaluation was 1 h and 50 ± 3 min. The concordance rate between evaluators on the way h and b measurements were taken and recorded was 98.5% and. 99.5%, respectively.

The mean area calculated for the right foot was 57.2 ± 15.23 mm^2^, and 54.6 ± 13.57 mm^2^ for the left foot (*p* > 0.05). Statistically significant differences were found in h, b, and area according to gender, these being smaller in women (*p* < 0.05).

The mean of b was between 4.4 ± 0.61 mm and 4.5 ± 0.70 mm, without statistically significant differences according to foot or evaluator (*p* > 0.05) when comparing Pre and Post measurements. There are no statistically significant differences either according to foot or evaluator when comparing Pre and Post measurements of h, with a mean of 12.2 ± 1.30 mm and 12.3 ± 1.33 mm (Table 1).

The concordance was analyzed, taking into account the differences between evaluators 1–2, 1–3, and 2–3, between Pre and Post measurements, foot (left and right), both for h and b measurements, obtaining in all cases an intraclass correlation coefficient of 1.00 (95%CI: 0.99–1.00), which is statistically significant (*p* < 0.05). Pearson’s correlation coefficient for h and b measurements and other variables can be seen in Table 2.

## 4. Discussion

Interevaluator measurements (concordance) and intraevaluator measurements (reliability) showed high concordance. The assessments, following the protocol, were easy to interpret, with short instructions, and were easy to understand and record, therefore obtaining high reliability [15]. All of this ensures that the evaluations carried out on the measurements of study (h, b) have been carefully verified, presenting excellent values of reliability.

Regarding the measurements of h and b, it has been observed that the intraevaluator measurements between Pre and Post did not show statistically significant differences (*p* > 0.05), and, therefore, they are reliable and constant over time.

These results highlight the usefulness and reliability of using US as a tool to measure the area of study related to the DTML. To our knowledge, studies assessing the reliability of US to take these measurements have not been performed. However, Santiago-Nuño et al. (2019) [4] conclude, in line with our results, that the use of US to measure the thickness, width, and length of the Spring Ligament is a good tool for evaluation, with an intraclass correlation coefficient between interevaluator measurements of 0.911–0.966, a value that approximates ours, whose interevaluator concordance reaches an intraclass correlation coefficient of 1.00. With this in mind, we can affirm that the two measurements taken with US of the area under the DTML are stable over time, and easily reproducible by any trained evaluator.

In the post-evaluation, full coincidences have been found, pairing the evaluators in every way possible [13]. It is possible to consider a learning bias derived from training the measurement process, and they might have unified their criteria (it is important to consider that between different sessions, evaluators discussed incidences observed with each other) [16].

Our dataset does not follow a parametric distribution; however, this does not affect the results obtained. It is expected that with a bigger sample size, a normal distribution could be obtained, or the possibility to treat the dataset as parametric applying the central limit theorem, or separating the sample in different and sufficiently representative groups.

We can conclude that h and b measurements are stable over time, and easily reproducible by any trained evaluator. Given these results, the aim of the study is verified, and thus, the measurement with ultrasound of the space under the deep transverse metatarsal ligament is reliable and repeatable.

As a proposal for the future, it is of interest to perform further research including other variables that could be instructive: foot size, dominant limb, profession, sports practice, age, weight, height, and gender, which may provide information on the non-parametric distribution of this analysis.

Considering previous studies, as in Kaminsky S et al. (1997) [17], and studies by Park YH et al. (2019) [18], ultrasound can be an effective tool for the specific diagnosis of Morton’s Neuroma. Other studies, such as Radwan et al. (2016) [19], compare ultrasound with magnetic resonance to study plantar fasciae, concluding that ultrasound provides a higher diagnostic value as compared to MRI, and is a more affordable option.

## 5. Conclusions

The ultrasound measurement of the area below the DTML and the third and fourth metatarsal heads may have diagnostic or predictive value for the development of Morton’s Neuroma.

## Figures and Tables

**Table 1:** Intra-rater reliability. Mean and standard deviation of h and b measurements (*p* < 0.05).

Evaluator	Foot	Phase	Base	Height
1	Left	Pre	4.4 ± 0.61	12.2 ± 1.29
	Right		4.5 ± 0.70	12.3 ± 1.33
	Left	Post	4.4 ± 0.61	12.2 ± 1.29
	Right		4.5 ± 0.70	12.3 ± 1.33
2	Left	Pre	4.4 ± 0.62	12.2 ± 1.29
	Right		4.5 ± 0.70	12.3 ± 1.33
	Left	Post	4.4 ± 0.61	12.2 ± 1.30
	Right		4.5 ± 0.70	12.3 ± 1.33
3	Left	Pre	4.4 ± 0.62	12.2 ± 1.29
	Right		4.5 ± 0.70	12.3 ± 1.32
	Left	Post	4.4 ± 0.61	12.2 ± 1.29
	Right		4.5 ± 0.70	12.3 ± 1.33

**Table 2:** Correlation between variables (*p* < 0.05). * European shoe size.

	Base	Height
Weight	0.637	0.629
Height	0.776	0.809
Shoe size *	0.870	0.903
